# Optimization of Boron Doped TiO_2_ as an Efficient Visible Light-Driven Photocatalyst for Organic Dye Degradation With High Reusability

**DOI:** 10.3389/fchem.2020.00172

**Published:** 2020-03-13

**Authors:** Pingping Niu, Guanghui Wu, Pinghua Chen, Huitao Zheng, Qun Cao, Hualin Jiang

**Affiliations:** ^1^Key Laboratory of Jiangxi Province for Persistent Pollutants Control and Resources Recycle, Nanchang, China; ^2^College of Environmental and Chemical Engineering, Nanchang Hangkong University, Nanchang, China

**Keywords:** TiO_2_, doping, boron, dye pollution, photocatalytic degradation

## Abstract

No visible light activity is the bottle neck for wide application of TiO_2_, and Boron doping is one of the effective way to broaden the adsorption edge of TiO_2_. In this study, several Boron doped TiO_2_ materials were prepared via a facile co-precipitation and calcination process. The B doping amounts were optimized by the degradation of rhodamine B (Rh B) under visible light irradiation, which indicated that when the mass fraction of boron is 6% (denoted as 6B-TiO_2_), the boron doped TiO_2_ materials exhibited the highest activity. In order to investigate the enhanced mechanism, the difference between B-doped TiO_2_ and bare TiO_2_ including visible light harvesting abilities, separation efficiencies of photo-generated electron-hole pairs, photo-induced electrons generation abilities, photo-induced charges transferring speed were studied and compared in details. h^+^ and ^·^${\text{O}}_{2}^{-}$ were determined to be the two main responsible active species in the photocatalytic oxidation process. Besides the high degradation efficiency, 6B-TiO_2_ also exhibited high reusability in the photocatalysis, which could be reused at least 5 cycles with almost no active reduction. The results indicate that 6B-TiO_2_ has high photocatalytic degradation ability toward organic dye of rhodamine B under visible light irradiation, which is a highly potential photocatalyst to cope with organic pollution.

## Introduction

Environmental problems are global issues and effect all of human kind. These issues and pressures increase in severity as society continues to develop at a very fast pace (Samanta et al., 2002; Shao et al., 2017; Chen et al., 2018; Chowdhary et al., 2018; Tian et al., 2018; Hong et al., 2020). Water pollution is one of the most serious environmental problems and attracts much attention (Wang and Yang, 2016; Jiang et al., 2019b; Kapelewska et al., 2019; Quesad et al., 2019; Wu et al., 2019; Zhao et al., 2019b). Organic dyes have been synthesized on a large scale and are widely applied in our daily lives, resulting in tons of dyes being discharged into the aqueous environment every year, causing many serious environmental problems (Sohni et al., 2019; Tu et al., 2019; Zhan et al., 2019; Zhou X. et al., 2019). Rhodamine B is a toxic alkaline cationic dye, which was used as a food additive, but has been forbidden due to its high carcinogenic potential (Wu et al., 2018; Lops et al., 2019; Tian et al., 2019; Guo et al., 2020). Furthermore, it can also cause other serious diseases such as visceral disease and red skin staining (Alcocer et al., 2018; Liu et al., 2019; Maria Magdalane et al., 2019). It is very difficult to degrade rhodamine B under natural conditions. Methods for the effective removal of rhodamine B are therefore of great importance.

In recent decades, photocatalysis has exhibited its high potential in waste water treatment, due to its inherent merits including low costs, renewability, being environment-friendly, and its high efficiency. TiO_2_ is a widely used photocatalyst because of its chemical stability, high redox reactivity, easy preparation, and low cost. However, it can not adsorb and use visible light because of its wide energy band gap. So only ~4% solar energy of ultraviolet light can be used by TiO_2_, and 45% solar energy of visible light can not be used (Jiang et al., 2018a, 2019a; Yin et al., 2018; Ahadi et al., 2019; Zhou F. et al., 2019; Komtchoua et al., 2020). In order to broaden the light adsorption of TiO_2_ to visible light, many efforts have been conducted (Zhao et al., 2019a; Zhang et al., 2020). Doing has attracted increasing interest in recent years (Jiang et al., 2018a; Kamaludin et al., 2019; Lu et al., 2019; Xiu et al., 2019; Yan et al., 2019).

In this study, boron was used to dope into TiO_2_ to prepare the photocatalyst of B-TiO_2_. B-TiO_2_ shows high photocatalytic degradation ability toward rhodamine B under visible light. The preparation conditions were optimized, and the structure and photocatalytic performance of B-TiO_2_ were carefully investigated. Based on the experimental results, the photocatalytic degradation mechanism was discussed. This study indicates that B-TiO_2_ has the potential to treat dye pollution through visible light irradiation.

## Experiment

### Materials

All the chemicals used in this study were of analytical pure grade. Boric acid and aqueous ammonia were purchased from Xilong Science Co., Ltd, China. Rhodamine B and tetrabutyl titanate were bought from the Aladdin reagent company, China. Other chemicals are all commercial. Deionized water (DI water) was used throughout the study. The materials were directly used without any treatment.

### Synthesis of B-TiO_2_

**Scheme 1:**
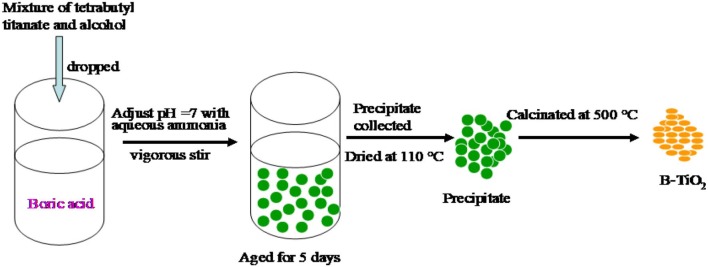
Synthesis scheme of B-TiO_2_.

0.1 mol tetrabutyl titanate was dissolved into 100 ml absolute alcohol to form a clean solution. Boric acid was dissolved into a solution containing 2 ml nitric acid, 50 ml absolute alcohol and 50 ml DI water. After that, the tetrabutyl titanate solution described above was dripped into the boric acid solution and vigorously stirred. At the same time, aqueous ammonia was dripped into the mixture described above to adjust the pH value to 7. The formation of precipitation was found in the process. After being aged for 5 days, the precipitation was separated and dried at 110°C. Finally, it was calcinated at 500°C to obtain the B doped TiO_2_ denoted as B-TiO_2_. The feeding amounts of boric acid were changed to obtain B-TiO_2_ with a B mass ratio of 0, 3, 6, 9, 12, and 15%, and are denoted as TiO_2_, 3B-TiO_2_, 6B-TiO_2_, 9B-TiO_2_, 12B-TiO_2_, and 15 B-TiO_2_, respectively. The methodology is shown in Scheme 1.

### Photocatalytic Degradation

Ten milligrams of photocatalyst was added into 25 ml rhodamine B solution at a concentration of 5 mg/L. The mixture was stirred in the dark for 30 min to obtain adsorption equilibrium. After that, the mixture was irradiated under visible light by a 500 W Xe lamp (Perfectlight, Beijing, China) with λ ≦ 400 nm cutoff, and sampled at determined intervals to examine the rhodamine B concentration in the suspension, which was determined by the adsorption at 552 nm.

### Characterization

The morphology of the sample was investigated by scanning electron microscopy (SEM) (Hitachi-4800, Japan) and a transmission electron microscope (TEM) (JEM-2100, Japan). An X-ray powder diffractometer (XRD, Rigaku III/B max, Cu Ka) was used to analyze the samples. The pH values of solutions were determined by a JENCO 6175 pH meter (Renshi electronics Co. Ltd. USA). Electrochemical impedance spectroscopy (EIS) and photocurrent response analysis were performed by CHI660C electrochemical workstation (Shanghai Chenhua, China). Photoluminescence (PL) spectra were recorded on a F-7000 fluorescence spectrophotometer (Hitachi, Japan). UV-vis diffuse-reflectance spectra (DRS) of samples were obtained on a UV-vis-NIR spectrometer (Lambda 900).

## Results and Discussion

### Optimization of B Doping Amount

**Figure 1:**
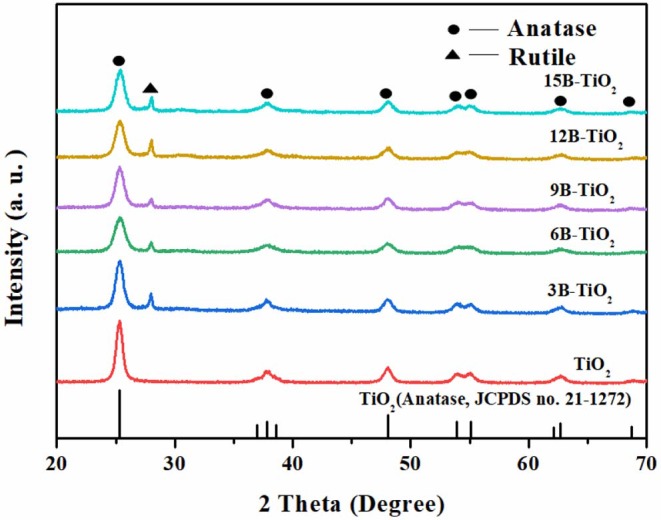
XRD spectrum of B-TiO_2_ and bare TiO_2_.

The bare TiO_2_ and B doping TiO_2_ were investigated by XRD analysis, and the results are shown in Figure 1. It can be seen that the samples show similar XRD characteristics. However, it is notable that there is a new peak at ~25.5°, which is attributable to rutile TiO_2_ (Wang et al., 2015; Warkhade et al., 2017), appears in the spectra of B doping TiO_2_, but is not the case in the spectrum of bare TiO_2_. This phenomenon indicates that under the experimental conditions of this study, the doping of B, no matter how much the doping amount is, can induce the pure anatase TiO_2_ to transform to mixed crystal phases of anatase and rutile (Cui et al., 2017). These results indicate that B has been successfully doped into the crystal lattice of TiO_2_.

**Figure 2:**
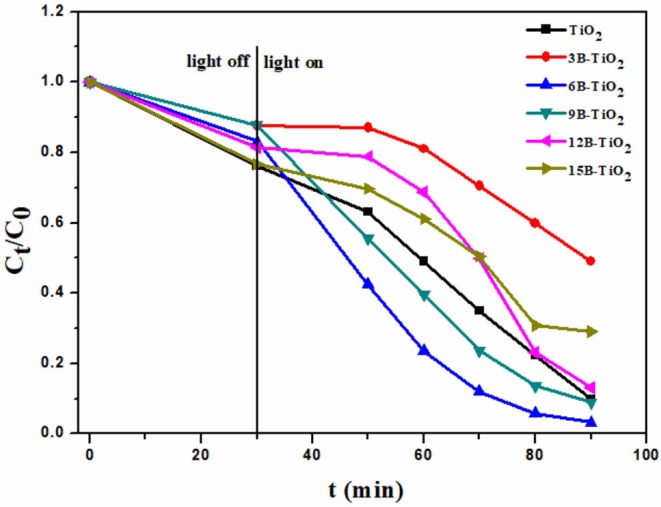
The photocatalytic degradation toward rhodamine B over B-TiO_2_ with different B doping amount.

All the samples, including the bare TiO_2_ and the B doping TiO_2_, were used to degrade rhodamine B under visible light irradiation. As shown in Figure 2, the degradation ability first increases with the B doping amount, and when the B doping amount reaches 6%, the degradation ability decreases with the rise of B doping amount. This phenomenon indicates that 6% of the B mass ratio is the optimal value. Thereafter, 6B-TiO_2_ was determined as the optimal sample, and 6% was determined as the optimal feeding amount of B in the preparation.

### Characterization of B Doping TiO_2_ Samples

**Figure 3:**
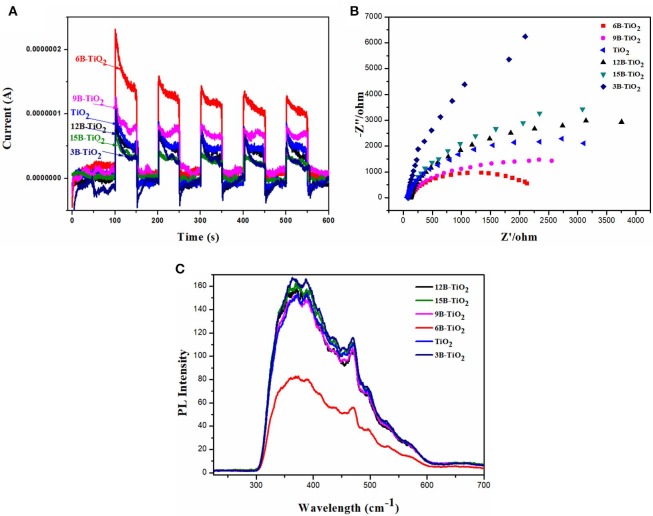
**(A)** Photocurrent response, **(B)** EIS, and **(C)** PL spectrum of bare TiO_2_ and B doping TiO_2_.

In order to investigate the mechanism of the enhanced photocatalytic performance of 6B-TiO_2_, the samples of bare TiO_2_ and B doping TiO_2_ were investigated by photocurrent response, EIS and PL spectrum, and the results are shown in Figure 3. It can be seen in Figure 3A that 6B-TiO_2_ shows highest photocurrent response, indicating that more electrons can be generated in 6B-TiO_2_ by visible light irradiation (Hu et al., 2019; Murali et al., 2019; Wang et al., 2019). In the EIS analysis (Figure 3B), 6B-TiO_2_ exhibits a semicircle of the EIS Nyquist plot with the smallest radius, indicating the smallest interfacial charge transference impedance as compared to those of bare TiO_2_ and other B doping TiO_2_ (Dai et al., 2015; Zou et al., 2016; Manwar et al., 2019). As shown in Figure 3C, 6B-TiO_2_ indicates the lowest photoluminescence intensity, which suggests that 6B-TiO_2_ has the lowest recombination rate of electron-hole pairs (Cai et al., 2019; Huang et al., 2019; Yuan et al., 2019). It can be found from the above analysis, that the most electrons can be generated by visible light in 6B-TiO_2_, and the charge carriers can transfer in 6B-TiO_2_ with the lowest impedance and recombination rate. All of these characteristic can favor the subsequent photocatalytic reaction, so there is no doubt that 6B-TiO_2_ exhibits the best photocatalytic performance as compared to bare TiO_2_ and other B doping TiO_2_.

**Figure 4:**
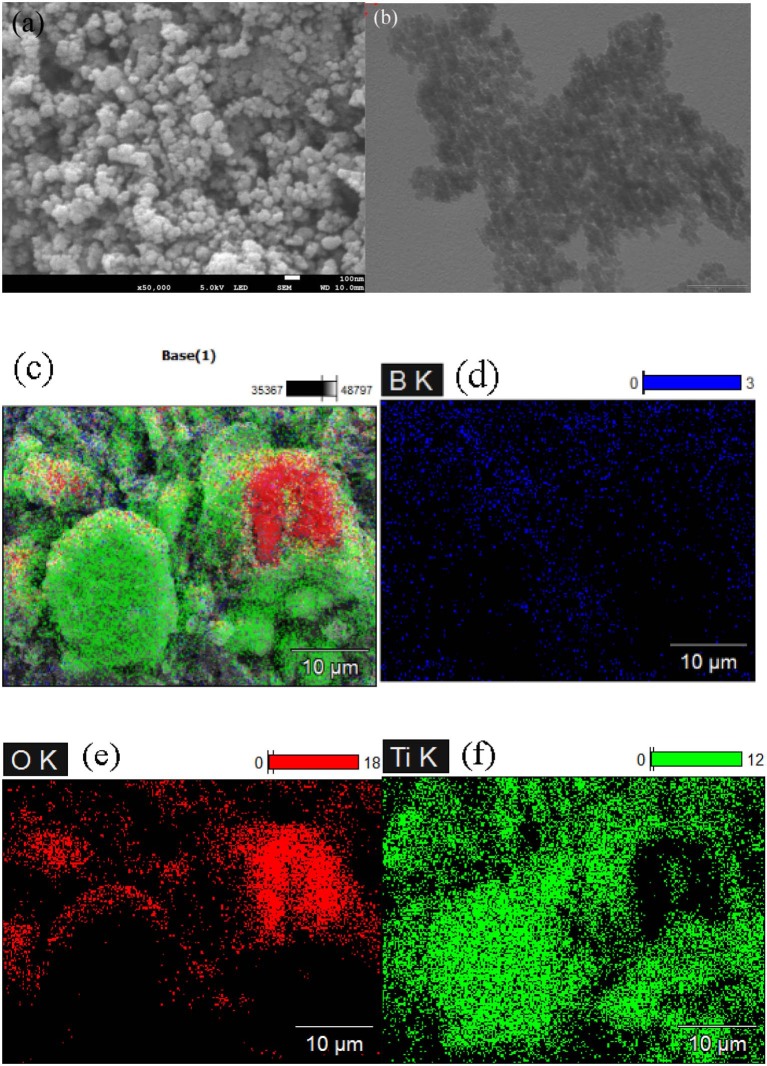
**(a)** SEM and **(b)** TEM imagines of 6B-TiO_2_; **(c–f)** the elemental mapping of 6B-TiO_2_.

The morphology of 6B-TiO_2_ was investigated by SEM and TEM. As shown in Figures 4a,b, 6B-TiO_2_ exhibits nano spheral morphology with a litter aggregation. Elemental mapping indicates that elements of B, O, and Ti homogeneously distribute on the surface of 6B-TiO_2_, confirming the successful doping of B (Figures 4c–f).

**Figure 5:**
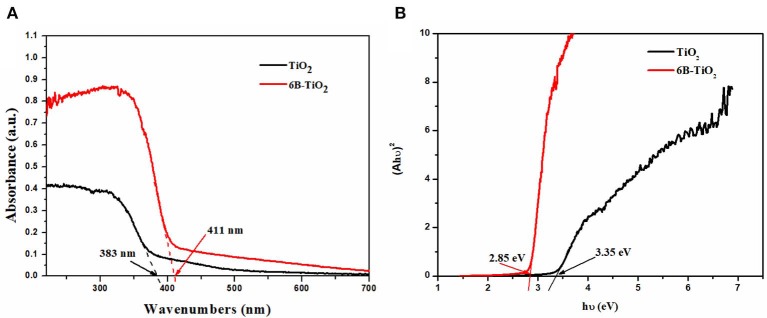
**(A)** Uv-vis DRS and **(B)** Tauc plot of bare TiO_2_ and 6B-TiO_2_.

The bare TiO_2_ and 6B-TiO_2_ were investigated by DRS, and the results are shown in Figure 5A. It can be seen that the light adsorption edge of bare TiO_2_ is about 383 nm, indicating no visible light adsorption activity. As B was doped to form 6B-TiO_2_, the light adsorption edge significantly red shift to about 411 nm, which indicates that 6B-TiO_2_ can adsorb visible light. The band gap energies of the two samples were calculated by Tauc plot according to the DRS results by Jiang et al. (2018b); Kato et al. (2019); Khan et al. (2019), and are shown in Figure 5B. As one can see, bare TiO_2_ has a wide band gap of 3.35 eV, which is too high to be excited by visible light. However, after B doping, the band gap was narrowed to 2.85 eV, and can be excited to produce electrons by visible light. The results clearly show that B doping significantly narrows the band gap of TiO_2_, and broadens the light adsorption edge to a visible light range.

### Investigation of the Active Species in the Photocatalytic Degradation

**Figure 6:**
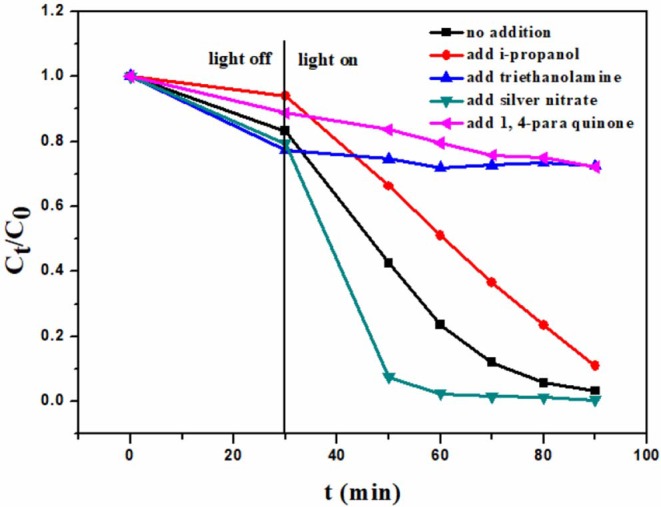
Photocatalytic degradation of rhodamine B over 6B-TiO_2_ in the presence of different scavengers.

In order to study the photocatalytic mechanism, the potential active species in the photocatalytic degradation course were investigated. There are usually four active species involved in the photocatalytic degradation course. They are ^·^${\text{O}}_{2}^{-}$, ^·^OH, e^−^, and h^+^. 0.05 mmol different scavengers were added, respectively, in the photocatalytic degradation system, and other conditions were the same as described in “2.3 Photocatalytic degradation.” The scavengers are i-propanol (^·^OH scavenger), triethanolamine (h^+^ scavenger), 1, 4-Benzoquinone (^·^${\text{O}}_{2}^{-}$), and AgNO_3_ (e^−^ scavenger) (Asmus et al., 1967; Zhang et al., 2013; Huyen et al., 2018). It can be seen in Figure 6 that h^+^ and ^·^${\text{O}}_{2}^{-}$ play the most important roles in the degradation of rhodamine B, because the addition of the scavengers for these two active species can dramatically decrease the degradation capacity. One can also find that ^·^OH takes part in the degradation, because the addition of i-propanol can impress the degradation too. h^+^, ^·^${\text{O}}_{2}^{-}$ and ^·^OH are three highly oxidative species. The results indicate that their high oxidative activities may be used to degrade rhodamine B in this photocatalysis course. It is notable that the addition of AgNO_3_ can promote the degradation. The reason is that AgNO_3_ can consume e^−^ as an e^−^ scavenger, which depress the recombination of e^−^-h^+^ pairs, and enhance the photocatalytic ability.

### Investigation of Reusability

**Figure 7:**
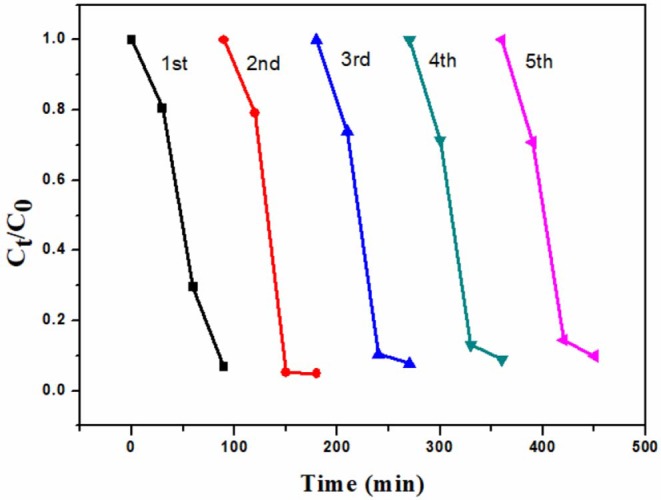
The reusability of 6B-TiO_2_.

In order to evaluate of the reusability of 6B-TiO_2_, the used 6B-TiO_2_ was collected and washed wth DI water. After being dried, it was used in the photocatalytic degradation of rhodamine B again, and the experimental conditions are the same as descried in “2.3 Photocatalytic degradation” with little modification of sampling time. As one can see in Figure 7, 6B-TiO_2_ can be continuously used to effectively degrade rhodamine B at least in five cycles.

## Conclusion

B doping TiO_2_ were successfully prepared in this study, and 6B-TiO_2_ was determined as the optimal B doping amount. 6B-TiO_2_ shows the best photocurrent response ability, fastest charge transference speed and lowest recombination rate of e^−^-h^+^ pairs, which significantly enhances its photocatalytic performance. B doping significantly narrows the band gap of TiO_2_, and therefore broaden the light adsorption edge to visible light range. h^+^ and ^·^${\text{O}}_{2}^{-}$ are the most important active species in the photocatalytic degradation, and ^·^OH is also involved in the degradation. 6B-TiO_2_ shows high reusability, which can be effectively used in at least five degradation cycles. 6B-TiO_2_ is a potential photocatalyst with visible light responsible ability, which can be used to effectively treat dye pollution.

## Data Availability Statement

All datasets generated for this study are included in the article/supplementary material.

## Author Contributions

PN did the experiments. PC and HJ designed the experiments. GW, HZ, and QC were devoted to the discussion and analysis.

### Conflict of Interest

The authors declare that the research was conducted in the absence of any commercial or financial relationships that could be construed as a potential conflict of interest.

## References

[B1] Ahadi S. Moalej N. S. Sheibani S. (2019). Characteristics and photocatalytic behavior of Fe and Cu doped TiO_2_ prepared by combined sol-gel and mechanical alloying. Solid State Sci. 96:105975 10.1016/j.solidstatesciences.2019.105975

[B2] Alcocer S. Picos A. Uribe A. R. Pérez T. Peraltahernández J. M. (2018). Comparative study for degradation of industrial dyes by electrochemical advanced oxidation processes with BDD anode in a laboratory stirred tank reactor. Chemosphere 205, 682–689. 10.1016/j.chemosphere.2018.04.155 29729622

[B3] Asmus K. D. Cercek B. Ebert M. Henglein A. Wigger A. (1967). Pulse radiolysis of nitrobenzene solutions. Trans. Faraday Soc. 63, 2435–2441. 10.1039/tf9676302435

[B4] Cai S. L. Lu L. Wu W. P. Wang J. Sun Y. C. Ma A. Q. (2019). A new mixed ligand based Cd(II) 2D coordination polymer with functional sites: photoluminescence and photocatalytic properties. Inorg. Chim. Acta. 484, 291–296. 10.1016/j.ica.2018.09.066

[B5] Chen P. Wang T. Xiao Y. Tian E. Wang W. Yule Z. (2018). Efficient fluoride removal from aqueous solution by synthetic Fe-Mg-La tri-metal nanocomposite and the analysis of its adsorption mechanism. J. Alloy. Compd. 738, 118–129. 10.1016/j.jallcom.2017.12.142

[B6] Chowdhary P. Raj A. Bharagava R. N. (2018). Environmental pollution and health hazards from distillery wastewater and treatment approaches to combat the environmental threats: a review. Chemosphere 194, 229–246. 10.1016/j.chemosphere.2017.11.163 29207355

[B7] Cui Y. Ma Q. Deng X. Meng Q. Cheng X. Xie M. (2017). Fabrication of Ag-Ag_2_O/reduced TiO_2_ nanophotocatalyst and its enhanced visible light driven photocatalytic performance for degradation of diclofenac solution. Appl. Catal. B Environ. 206, 136–145. 10.1016/j.apcatb.2017.01.014

[B8] Dai Z. Qin F. Zhao H. Tian F. Liu Y. Chen R. (2015). Time-dependent evolution of the Bi_3.64_Mo_0.36_O_6.55_/Bi_2_MoO_6_ heterostructure for enhanced photocatalytic activity via the interfacial hole migration. Nanoscale 7, 11991–11999. 10.1039/C5NR02745D 26108795

[B9] Guo N. Liu H. Fu Y. Hu J. (2020). Preparation of Fe_2_O_3_ nanoparticles doped with In_2_O_3_ and photocatalytic degradation property for rhodamine B. Optik 201:163537 10.1016/j.ijleo.2019.163537

[B10] Hong S. M. Yoon H. J. Choi Y. Cho Y. Z. Mun S. Pol V. G. (2020). Solving two environmental problems simultaneously: scalable production of carbon microsheets from structured packing peanuts with tailored microporosity for efficient CO_2_ capture. Chem. Eng. J. 379:122219 10.1016/j.cej.2019.122219

[B11] Hu T. Wang W. Han D. Dong W. (2019). Enhanced photocurrent and photocatalytic degradation of methyl orange in cobalt hydroxide loaded titanium dioxide film. AIP Adv. 9:055122 10.1063/1.5098399

[B12] Huang Y. Qin J. Fan Z. Wei D. Seo H. J. (2019). Photoenergy conversion behaviors of photoluminescence and photocatalysis in silver-coated LiBaPO_4_:Eu^2+^. Inorg. Chem. 58, 13161–13169. 10.1021/acs.inorgchem.9b02037 31498607

[B13] Huyen T. T. T. Chi T. T. K. Dung N. D. Kosslick H. Liem N. Q. (2018). Enhanced photocatalytic activity of {110}-faceted TiO_2_ rutile nanorods in the photodegradation of hazardous pharmaceuticals. Nanomaterials 8:276 10.3390/nano8050276 PMC5977290 29693630

[B14] Jiang H. Li M. Liu J. Li X. Tian L. Chen P. (2018a). Alkali-free synthesis of a novel heterostructured CeO_2_-TiO_2_ nanocomposite with high performance to reduce Cr(VI) under visible light. Ceram. Int. 44, 2709–2717. 10.1016/j.ceramint.2017.10.225

[B15] Jiang H. Li X. Li M. Niu P. Wang T. Chen D. (2019a). A new strategy for triggering photocatalytic activity of cytrochrome P450 by coupling of semiconductors. Chem. Eng. J. 358, 58–66. 10.1016/j.cej.2018.09.199

[B16] Jiang H. Li X. Tian L. Wang T. Wang Q. Pingping N.. (2019b). Defluoridation investigation of yttrium by laminated Y-Zr-Al tri-metal nanocomposite and analysis of the fluoride sorption mechanism. Sci. Total Environ. 648, 1342–1353. 10.1016/j.scitotenv.2018.08.258 30340280

[B17] Jiang H. Liu J. Li M. Tian L. Ding G. Chen P. (2018b). Facile synthesis of C-decorated Fe, N co-doped TiO_2_ with enhanced visible-light photocatalytic activity by a novel co-precursor method. Chin. J. Catal. 39, 747–759. 10.1016/S1872-2067(18)63038-4

[B18] Kamaludin R. Puad A. S. M. Othman M. H. D. AbdulKadir S. H. S. Harun Z. (2019). Incorporation of N-doped TiO_2_ into dual layer hollow fiber (DLHF) membrane for visible light-driven photocatalytic removal of reactive black 5. Polym. Test. 78:105939 10.1016/j.polymertesting.2019.105939

[B19] Kapelewska J. Kotowska U. Karpinska J. Astel A. Zielinski P. Suchta J. (2019). Water pollution indicators and chemometric expertise for the assessment of the impact of municipal solid waste landfills on groundwater located in their area. Chem. Eng. J. 359, 790–800. 10.1016/j.cej.2018.11.137

[B20] Kato K. Vaucher S. Hoffmann P. Xin Y. Shirai T. (2019). A novel single-mode microwave assisted synthesis of metal oxide as visible-light photocatalyst. Mater. Lett. 235, 125–128. 10.1016/j.matlet.2018.09.132

[B21] Khan M. S. Diao Z. Osada M. Shen S. (2019). Nitrogen doped ultrathin calcium/sodium niobate perovskite nanosheets for photocatalytic water oxidation. Sol. Energy Mater. Sol. Cells 205:110283 10.1016/j.solmat.2019.110283

[B22] Komtchoua S. Delegan N. Dirany A. Drogui P. Robert D. Khakani M. A. E. (2020). Photo-electrocatalytic oxidation of atrazine using sputtured deposited TiO2: WN photoanodes under UV/visible light. Cataly. Today 340, 323–333. 10.1016/j.cattod.2019.04.067

[B23] Liu N. Jing C. Li Z. Huang W. Gao B. You F. (2019). Effect of synthesis conditions on the photocatalytic degradation of rhodamine B of MIL-53(Fe). Mater. Lett. 237, 92–95. 10.1016/j.matlet.2018.11.079

[B24] Lops C. Ancona A. Cesare K. D. Dumontel B. Garino N. Canavese G.. (2019). Sonophotocatalytic degradation mechanisms of rhodamine B dye via radicals generation by micro- and nano-particles of ZnO. Appl. Cata. B Environ. 243, 629–640. 10.1016/j.apcatb.2018.10.078 30886458 PMC6420045

[B25] Lu X. Li X. Chen F. Chen Z. Qian J. Zhang Q. (2019). Biotemplating synthesis of N-doped two-dimensional CeO_2_-TiO_2_ nanosheets with enhanced visible light photocatalytic desulfurization performance. J. Alloy. Comp. 815:152326 10.1016/j.jallcom.2019.152326

[B26] Manwar N. R. Jain S. L. Rayalu S. Labhasetwar N. K. (2019). Solar energy conversion: Pt-doped mesoporous ceria as an efficient electro-photocatalyst for hydrogen production from water splitting. Mater. Today Proc. 17, 277–287. 10.1016/j.matpr.2019.06.431

[B27] Maria Magdalane C. Kaviyarasu K. Priyadharsini G. M. A. Bashir A. K. H. Mayedwa N. Matinise N. (2019). Improved photocatalytic decomposition of aqueous rhodamine-B by solar light illuminated hierarchical yttria nanosphere decorated ceria nanorods. J. Mater. Res. Technol. 8, 2898–2909. 10.1016/j.jmrt.2018.11.019

[B28] Murali A. Sarswat P. K. Sohn H. Y. (2019). Enhanced photocatalytic activity and photocurrent properties of plasma-synthesized indium-doped zinc oxide nanopowder. Mater. Today Chem. 11, 60–68. 10.1016/j.mtchem.2018.10.007

[B29] Quesad H. B. Baptista A. T. A. Cusioli L. F. Seibert D. Bezerra C. O. Bergamasco R. (2019). Surface water pollution by pharmaceuticals and an alternative of removal by low-cost adsorbents: a review. Chemosphere 222, 766–780. 10.1016/j.chemosphere.2019.02.009 30738319

[B30] Samanta S. K. Singh O. V. Jain R. K. (2002). Polycyclic aromatic hydrocarbons: environmental pollution and bioremediation. Trends Biotechnol. 20, 243–248. 10.1016/S0167-7799(02)01943-1 12007492

[B31] Shao J. Sheng W. Wang M. Li S. Chen J. Zhang Y. (2017). *In situ* synthesis of carbon-doped TiO_2_ single-crystal nanorods with a remarkably photocatalytic efficiency. Appl. Catal. B Environ. 209, 311–319. 10.1016/j.apcatb.2017.03.008

[B32] Sohni S. Hashim R. Nidaullah H. Lamaming J. Sulaiman O. (2019). Chitosan/nano-lignin based composite as a new sorbent for enhanced removal of dye pollution from aqueous solutions. Int. J. Biol. Macromol. 132, 1304–1317. 10.1016/j.ijbiomac.2019.03.151 30922916

[B33] Tian J. Shao Q. Zhao J. Pan D. Dong M. Jia C.. (2019). Microwave solvothermal carboxymethyl chitosan templated synthesis of TiO_2_/ZrO_2_ composites toward enhanced photocatalytic degradation of rhodamine B. J. Colloid Interf. Sci. 541, 18–29. 10.1016/j.jcis.2019.01.069 30682590

[B34] Tian L. Jiang H. L. Chen P. H. Wang Q. Niu P. P. Shi Y. M. (2018). A novel GO/PNIPAm hybrid with two functional domains can simultaneously effectively adsorb and recover valuable organic and inorganic resources. Chem. Eng. J. 343, 607–618. 10.1016/j.cej.2018.03.015

[B35] Tu H. Li D. Yi Y. Liu R. Wu Y. Dong X. (2019). Incorporation of rectorite into porous polycaprolactone/TiO_2_ nanofibrous mats for enhancing photocatalysis properties towards organic dye pollution. Composite. Comm. 15, 58–63. 10.1016/j.coco.2019.06.006

[B36] Wang B. Zhang G. Sun Z. Zheng S. Frost R. L. (2015). A comparative study about the influence of metal ions (Ce, La and V) doping on the solar-light-induced. J. Environ. Chem. Eng. 3, 1444–1451. 10.1016/j.jece.2015.05.007

[B37] Wang Q. Yang Z. (2016). Industrial water pollution, water environment treatment, and health risks in China. Environ. Epidemiol. 218, 358–365. 10.1016/j.envpol.2016.07.011 27443951

[B38] Wang Y. Shen L. Wang Y. Hou B. Gibson G. N. Poudel N.. (2019). Hot electron-driven photocatalysis and transient absorption spectroscopy in plasmon resonant grating structures. Faraday Discuss. 214, 325–339. 10.1039/C8FD00141C 31049541

[B39] Warkhade S. K. Gaikwad G. S. Zodape S. P. Pratap U. Maldhure A. V. Wankhade A. V. (2017). Low temperature synthesis of pure anatase carbon doped titanium dioxide: an efficient visible light active photocatalyst. Mat. Sci. Semicon. Proc. 63, 18–24. 10.1016/j.mssp.2017.01.011

[B40] Wu X. Li H. Su J. Zhang J. Feng Y. Jia Y. (2019). Full spectrum responsive In_2.77_S_4_/WS_2_ p-n heterojunction as an efficient photocatalyst for Cr(VI) reduction and tetracycline oxidation. Appl. Surf. Sci. 473, 992–1001. 10.1016/j.apsusc.2018.12.219

[B41] Wu X. Sun Y. Li H. Wang Y. Zhang C. Zhang J. (2018). *In-situ* synthesis of novel p-n heterojunction of Ag_2_CrO_4_-Bi_2_Sn_2_O_7_ hybrids for visible-light-driven photocatalysis. J. Alloy Compd. 740, 1197–1203. 10.1016/j.jallcom.2018.01.100

[B42] Xiu Z. Guo M. Zhao T. Pan K. Xing Z. Zhenzi L. (2019). Recent advances in Ti^3+^ self-doped nanostructured TiO_2_ visible light photocatalysts for environmental and energy applications. Chem. Eng. J. 382:123011 10.1016/j.cej.2019.123011

[B43] Yan J. Zha J. Hao L. Hu Y. Liu T. Guan S. (2019). Low-temperature S-doping on N-doped TiO_2_ films and remarkable enhancement on visible-light performance. Mater. Res. Bull. 120:110594 10.1016/j.materresbull.2019.110594

[B44] Yin Z. Qiu S. Chen W. Li H. Cheng L. Cao S. (2018). Highly photocatalytic activity from tri-modified TiO_2_ hollow spheres. Mater. Lett. 214, 202–204. 10.1016/j.matlet.2017.11.123

[B45] Yuan H. Xu M. Luo K. Hu W. (2019). Relationships between defect-related photoluminescence and photocatalytic activity of (F, Na)-codoped ZnO nanocrystals. Ceram. Int. 45, 16694–16697. 10.1016/j.ceramint.2019.05.136

[B46] Zhan Y. Guan X. Ren E. Lin S. Lan J. (2019). Fabrication of zeolitic imidazolate framework-8 functional polyacrylonitrile nanofibrous mats for dye removal. J. Polym. Res. 26:145 10.1007/s10965-019-1806-5

[B47] Zhang S. Yi J. Chen J. Yin Z. Tang T. Wei W. (2020). Spatially confined Fe_2_O_3_ in hierarchical SiO_2_@TiO_2_ hollow sphere exhibiting superior photocatalytic efficiency for degrading antibiotics. Chem. Eng. J. 380:122583 10.1016/j.cej.2019.122583

[B48] Zhang Y. Zhang N. Tang Z. R. Xu Y. J. (2013). Identification of Bi_2_WO_6_ as a highly selective visible-light photocatalyst toward oxidation of glycerol to dihydroxyacetone in water. Chem. Sci. 4, 1820–1824. 10.1039/c3sc50285f

[B49] Zhao S. Chen J. Liu Y. Jiang Y. Jiang C. Yin Z. (2019a). Silver nanoparticles confined in shell-in-shell hollow TiO2 manifesting efficiently photocatalytic activity and stability. Chem. Eng. J. 367, 249–259. 10.1016/j.cej.2019.02.123

[B50] Zhao S. Zhu H. Wang H. Rassu P. Wang Z. Song P.. (2019b). Free-standing graphene oxide membrane with tunable channels for efficient water pollution control. J. Hazard. Mater. 366, 659–668. 10.1016/j.jhazmat.2018.12.055 30580140

[B51] Zhou F. Yan C. Sun Q. Komarneni S. (2019). TiO_2_/Sepiolite nanocomposites doped with rare earth ions: preparation, characterization and visible light photocatalytic activity. Micropor. Mesopor. Mater. 274, 25–32. 10.1016/j.micromeso.2018.07.031

[B52] Zhou X. Zhou Y. Liu J. Song S. Sun J. Zhu G.. (2019). *Study* on the pollution characteristics and emission factors of PCDD/Fs from disperse dye production in China. Chemosphere 228, 328–334. 10.1016/j.chemosphere.2019.04.136 31039539

[B53] Zou J. Wang L. Luo J. Nie Y. Xing Q. Luo X. (2016). Synthesis and efficient visible light photocatalytic H_2_ evolution of a metal-free g-C_3_N_4_/graphene quantum dots hybrid photocatalyst. Appl. Catal. B Environ. 193, 103–109. 10.1016/j.apcatb.2016.04.017

